# Effect of bleaching agents on hardness, surface roughness and color parameters of dental enamel

**DOI:** 10.4317/jced.56913

**Published:** 2020-07-01

**Authors:** Ana-Caroline-Godoy de Carvalho, Terezinha-Fatima de Souza, Priscila-Christiane-Suzy Liporoni, Eliane-Cristina-Gava Pizi, Larissa-Sgarbosa-de Araújo Matuda, Anderson Catelan

**Affiliations:** 1 Undergraduate Student, School of Dentistry, Faculty of Health Sciences, University of Western São Paulo, Presidente Prudente, SP, Brazil; 2 Graduate Student, Graduate Program in Dentistry, Faculty of Health Sciences, University of Western São Paulo, Presidente Prudente, SP, Brazil; 3 Assistant Professor, Department of Dentistry, University of Taubaté, Taubaté, SP, Brazil; 4 Assistant Professor, Graduate Program in Dentistry, Faculty of Health Sciences, University of Western São Paulo, Presidente Prudente, SP, Brazil; 5 Assistant Professor, School of Dentistry, Faculty of Health Sciences, University of Western São Paulo, Presidente Prudente, SP, Brazil

## Abstract

**Background:**

In this study was evaluated the effect of carbamide peroxide (CP) and hydrogen peroxide (HP) in different concentrations on hardness, roughness, and color parameters (color change - ΔE, lightness - ΔL, and yellow-blue axis - Δb) of bovine teeth.

**Material and Methods:**

Fifty square dental blocks (7 x 7 x 2 mm) were submitted to initial readings of Knoop hardness, surface roughness (Ra), and color parameters. Specimens were divided into 5 groups (n = 10): control group was kept in artificial saliva during the experimental period; CP 20% was bleached for 2 h daily for 14 days, HP 9.5% was bleached for 30 min daily for 14 days, HP 38% the bleaching gel was applied for 15 min, gel was removed and it was reapplied for two more times, the bleaching session was repeated for another 2 times every 7 days, totaling three sessions, and in CP 45% three sessions of 30 min each were performed of 7 in 7 days. All groups after the bleaching procedures had the bleaching gel removed, washed, and kept in artificial saliva. At the end of bleaching treatment, the hardness, roughness, and color parameters (ΔE, ΔL, and Δb) were re-evaluated. Data were analyzed by ANOVA, Tukey, and Dunnett tests (α = 0.05).

**Results:**

Bleaching promoted a reduction in hardness, the CP 45% showed the lowest hardness and the CP 20% the highest, the HP 9.5% and HP 38% showed intermediate values of hardness. Bleaching agents did not affect the roughness. CP 20% and HP 38% promoted the highest values of ΔE and higher reduction of yellowish tone of tooth. Lightness increase after bleaching treatment for all groups.

**Conclusions:**

All the bleaching agents tested showed effectiveness, but with reduced hardness.

** Key words:**Carbamide peroxide, hydrogen peroxide, tooth whitening, hardness, roughness.

## Introduction

Darkened teeth have been the cause of recurrent dissatisfaction and with this, dental bleaching has been increasingly widespread and accepted among patients, as it is considered an effective, safe, and minimally invasive procedure (1-4). In order to meet the demand of esthetic dentistry, manufacturers of dental products constantly develop new bleaching agents with different concentrations and application protocols, which seeks a faster and more satisfying bleaching effect, making the choice of bleaching product an increasingly difficult choice (2,5,6).

Bleaching agents commonly used are hydrogen peroxide (HP) and carbamide peroxide (CP) which can be found in different concentrations, and they can be applied at home or in-office, with variation in contact time of bleaching gel with dental tissue. (1,3,4,7-11). Approximately one third of CP decomposes to HP, which is the active agent of bleaching (1,4,8,9,12). Peroxides are unsTable substances that, in contact with dental tissues and moisture, release free radicals such as oxygen ions, which have the ability to diffuse through enamel and dentin and cause the oxidation of pigmented molecules that cause the tooth structure to darken (4,6,8,11-14). Home-use bleaching agents have been widely used in recent years, but some patients do not want to use bleaching trays or wait a few weeks to see the results of the bleaching, so these patients seek the in-office bleaching treatment that has more immediate results (2,3,11,15). On the other hand, this slower whitening effect in home bleaching promotes greater color stability in long term when compared to in-office technique (16). In-office bleaching has advantage to avoid the exposure of soft tissues to bleaching agent and ingestion (4,10,11).

CP was initially proposed for home use in low concentration trays. However, it was introduced the bleaching in waiting room or reception, this technique consists in use of trays with high concentration CP in dental office waiting area, under professional supervision. Bleaching gels with high concentration of peroxide and shorter contact time can be less harmful to enamel when compared to treatments with low concentration gels for longer application time (1,4,8). Studies (1,4) have related that high concentration of CP and HP bleaching gels used for a shorter time did not show negative effects on enamel, while low concentration gels of peroxides caused changes on enamel surface such as decrease in its hardness and increased surface roughness. However, other studies (17,18) related that all the bleaching agents reduced the enamel hardness and increased the surface roughness, and that changes caused by products with high concentrations do not differ substantially from those caused by low concentrations, these discrepancies can occur due to different models of applied studies (4,9).

So, although bleaching products promote efficacy on color change, making teeth lighter, the possible negative effects on enamel are still controversial in literature (4). It is known that enamel can show changes in its chemical composition and morphological changes due to changes in inorganic and organic composition caused by peroxide-based bleaching agents, and that free radicals generated during bleaching procedure can increase porosity, because it reacts non-selectively with organic structures of dental tissues. In addition, the bleaching products can generate gingival irritation and increased sensitivity as side effects (1,6,11-13,18,19).

Bleaching effectiveness as well as the changes caused on enamel surface and side effects depends mainly on relationship established between the concentration of gel and its time of use, and may also be influenced by diffusion capacity of bleaching agent and its pH (4-6,9,11,14). The pH can change not only the bleaching efficacy, but it can also reduce the hardness, increase the roughness and cause the wear of tooth enamel (6,13,20). In addition to factors associated with bleaching gels, there is also a concern with the patients’ diet, as excessive exposure to alcohol, food, and acidic drinks can generate deleterious effects on enamel surface when used frequently during the bleaching treatment and the literature shows the need to be cautious when indicating treatment for patients with dentin hypersensitivity (11-13,20).

Thus, it would be relevant to evaluate and compare the changes caused in teeth when subjected to different bleaching agents. Therefore, the aim in this study was to assess the Knoop hardness, surface roughness, and change in color parameters of dental specimens after bleaching treatment using CP- and HP-based gels in different concentrations. The null hypothesis was that bleaching agents would not affect the hardness, roughness, and color parameters.

## Material and Methods

-Experimental design

This is a randomized complete block arrangement laboratorial study that assessed the Knoop hardness, surface roughness, and change in color (ΔE), lightness (ΔL), and yellow-blue axis (Δb) of dental specimens, before and after bleaching procedure using CP and HP. The studied factors were as follows: bleaching agent in five levels (no product - control, CP 20%, HP 9.5%, HP 38%, and CP 45%) and timespan study (before and after bleaching procedure).

-Specimen preparation

Eighty extracted bovine incisors were stored in 0.5% thymol solution. The roots were separated from crowns using a diamond disc (KG Sorensen, Cotia, SP, Brazil) mounted in a low speed handpiece under water cooling. Then, buccal faces of crowns were sectioned in mesio-distal and buccal-lingual directions to obtain a square dental block (7 mm x 7 mm x 2 mm) using a metallographic saw (Isomet 1000; Buehler Inc., Lake Bluff, IL, USA). The enamel surface was polished for 30 s with 600-, 1200-, and 2500-grit silicon carbide abrasive papers using a polishing machine (APL-4; Arotec, Cotia, SP, Brazil) and subjected to cleaning in an ultrasonic cleaner containing distilled water for 5 min (Cristófoli, Campo Mourão, PR, Brazil).

Final polishing was carried out with diamond solution (MetaDi Supreme; Buehler Inc.) of ½ and ¼ granulation and felt disc for 30 s using the polishing machine; between the solutions and at end of process the specimens were cleaned ultrasonically for 5 min. Dental blocks were immersed in artificial saliva for 30 days to standardize the mineral content, changed daily (19). Then, blocks were embedded in polystyrene resin Piraglass, Piracicaba, SP, Brazil) with buccal enamel surface exposed.

-Knoop hardness

After storage in saliva, hardness was measured on top surface of each specimen using a microhardness tester (HMV-G20ST; Shimadzu Corp., Tokyo, Japan). Three measurements were performed by same operator under a load of 25 g for 5 s. An indentation was performed in middle of specimen, one to right and one to left with 100 µm from central location. Knoop hardness number (KHN) of each specimen was considered the arithmetic mean of three indentations (18). Fifty dental blocks with a hardness between 290-350 Kgf/mm2 were selected.

-Surface roughness

Surface roughness was evaluated using a rugosimeter (Hommel-Etamic w10; Villingen-Schwenningen, Germany). Roughness profile used was the mean roughness (Ra), which represents the arithmetic mean between recorded peaks and valleys. Readings were performed at a speed of 0.05 mm/s and cut-off of 0.25 mm. On each surface three readings were performed in different positions passing through the center of specimen, rotating the specimen approximately 120º (21). Roughness was obtained by arithmetic mean of three readings.

-Color measurement

The specimens were subjected to an initial chromatic analysis using the CIE L* a* b* system, established by Comission Internacionale de I’Eclairaga, using a reflectance spectrophotometer (VITA Easyshade Advance 4.0; Vident, Brea, CA, USA). The a* and b* axes have right angles and represent the color dimension (a*: green-red ratio; b*: blue-yellow ratio). Third axis (L*) represents the lightness, perpendicular to a* and b* planes (18).

-Bleaching treatment

After the initial readings of hardness, roughness, and color change, the specimens were divided into 5 groups (n = 10) and submitted to the bleaching procedures described below: (1) control group was kept in artificial saliva during the 14 days of experimental period; (2) CP 20% group (Opalescence PF 20%; Ultradent Inc., South Jordan, UT, USA) was bleached for 2 h daily for 14 days, for remaining 22 h of day the specimens were kept in artificial saliva at 37 ºC. For bleaching, 0.49 mL of bleaching agent (approximately 1 mm thick) was placed on surface of specimen and kept in a covered plastic pot containing gauze moistened at bottom and the set was kept in an incubator at 37ºC during bleaching period. At end of treatment, bleaching gel was removed with aid of flexible cotton swabs and finally washed in running water for 30 s, gently dried with absorbent paper and stored in saliva; (3) HP 9.5% (Poladay 9.5%; SDI, Bayswater, VIC, Australia) was bleached as previously described, however, the bleaching gel was applied for 30 min and after kept in saliva; (4) HP 38% group (Opalescence Boost 38%) the bleaching gel was applied for 15 min, after this period the gel was removed with the aid of flexible cotton swabs and bleaching agent was reapplied twice more. Then, specimens were cleaned, washed, and stored in saliva as previously described. Bleaching procedure was repeated twice more every 7 days, totaling three sessions; (5) CP 45% (Opalescence Quick PF 45%), three sessions of 30 min each were performed every 7 days. After bleaching session, specimens were cleaned, washed, and stored in saliva as previously described.

After 24 h of finished bleaching treatments the surface roughness and Knoop hardness were re-evaluated using the parameters previously described (18,21).

Color change (ΔE) was evaluated by difference between the coordinates obtained before and after bleaching, calculated from the formula: ΔE = [(ΔL*)2 + (Δa*)2 + (Δb*)2]½ (20). The change in lightness (ΔL) and the yellow-blue axis (Δb) were also calculated.

-Statistical analysis

Data were analyzed statistically at significance level of 5%. First, the normality and homogeneity of data were verified by the Kolmogorov-Smirnov and Levene tests, respectively. Then, Knoop hardness and surface roughness were subjected to two-way analysis of variance (ANOVA) for repeated measures, followed by Tukey’s test for multiple comparisons. For statistical analysis of parameters ΔE, ΔL and Δb one-way ANOVA was performed and Tukey’s test. Dunnett’s test was used to compare the experimental groups and control group (SPSS Version 20, IBM Corp., Armonk, NY, USA).

## Results

Bleaching treatment promoted a reduction in hardness values of all experimental groups (Table 1). CP 45% showed the lowest hardness and CP 20% the highest KHN values, HP 9.5% and HP 38% showed intermediate hardness values (*p* = 0.007) after treatment.

**Table 1:**
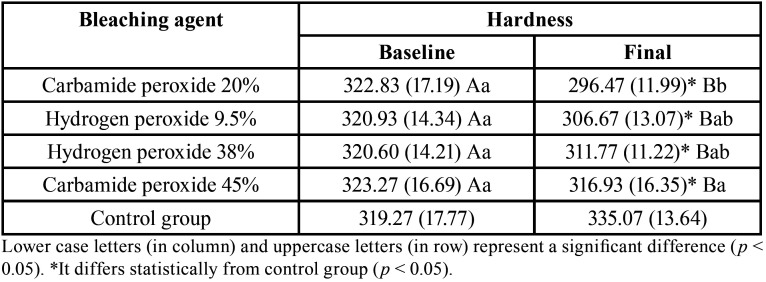
Knoop hardness (KgF/mm2) means (S.D.) according bleaching agent and timespan study.

The different bleaching agents did not cause an increase in enamel surface roughness values of experimental groups and these were statistically similar to control group, before and after the bleaching procedure (*p*> 0.05), as seen in Table 2.

**Table 2:**
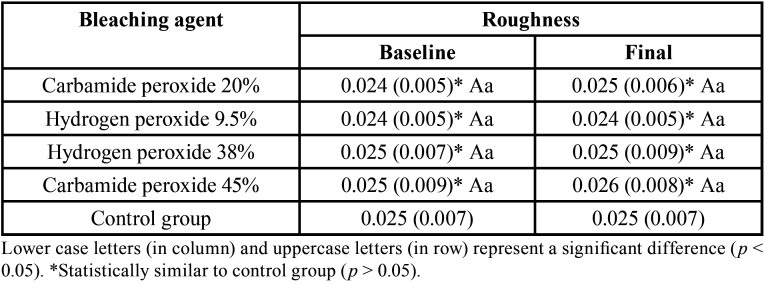
Surface roughness (Ra, μm) means (S.D.) according bleaching agent and timespan study.

ANOVA showed statistical difference for ΔE and Δb (*p* < 0.001 and *p* < 0.001, respectively), whereas for ΔL there was no significant difference between the experimental groups (*p* = 0.698). All experimental groups showed a significant color difference, with CP 20% and HP 38% promoting the highest values of ΔE and the lowest values of Δb, followed by HP 9.5% and CP 45%. In addition, all experimental groups had a higher ΔL compared to control group (Table 3).

**Table 3:**
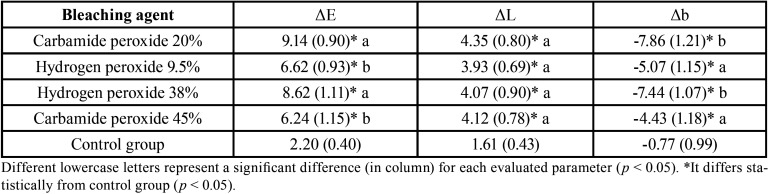
Color change (ΔE), lightness (ΔL), and blue-yellow axis (Δb) means (S.D.) according bleaching agent.

## Discussion

Morphological changes in enamel, caused by peroxide-based bleaching agents, have been reported due to changes in inorganic and organic composition, decreasing its hardness and increasing its surface roughness (1,4,19). In addition, during bleaching free radicals generated can increase the porosity on tooth surface, as it reacts non-selectively with the organic structures of dental tissues (1,13,19).

According to results obtained in this study, all bleaching agents tested reduced the enamel hardness, corroborating with previous studies (17-19,22,23). CP 20% showed the lowest Knoop hardness after the bleaching treatment, despite the lower HP concentration (~6.3%) among the evaluated products, this was the one that remained in contact with the teeth for the longest time (2 h daily for 14 days = 1680 min) could possibly explain this result. HP 9.5% (30 min daily for 14 days = 420 min) and HP 38% (3 sessions of 45 min each = 135 min) showed intermediate hardness reduction values, and CP 45% (~15% of HP) the lowest reduction (3 sessions of 30 min each = 90 min), this bleaching protocol was the one that the product stayed less time in contact with the tooth.

In addition to contact time of bleaching agent with enamel, the composition of bleaching gels, such as their concentration, pH, activators, and thickeners may be related to the hardness reduction (4,6,19). The pH can affect not only the bleaching efficiency but can also cause changes in enamel surface morphology and decreased hardness (13,19,20). Most of bleaching products available in dental market generally have a neutral or slightly acidic pH. However, after its application, acidification of medium usually occurs over time (24), which causes hardness reduction. However, clinically the exposure of teeth to saliva provides remineralization and normalization of enamel hardness after the bleaching treatment (25). During the bleaching procedure, the dental blocks were kept in artificial saliva and physical properties tested were re-evaluated 24 h after the end of last application of bleaching agent.

Studies (1,6,13,18,19) have reported an increase in enamel surface roughness, the HP reacts non-selectively with organic structures of dental tissues causing porosity in tooth. In addition to exposure of enamel prisms after bleaching treatment (25). Today it is known that the treatment of surfaces through polishing, the use of fluorides and remineralizing solutions considerably reduces the roughness of bleached enamel (6,20). However, in the present study, there was no difference in surface roughness values before and after bleaching, the possible changes in surface morphology were not significant to cause an increase on roughness measured by rugosimeter.

All bleaching products tested at different concentrations showed bleaching efficacy, attested by color change (ΔE), increased lightness (ΔL), and reduced values in blue-yellow axis (Δb). ΔE values between 3 to 8 are moderately visible and above 8 are extremely noticeable (13). Therefore, HP 9.5% and CP 45% showed a moderate change in color, and CP 20% and HP 38% showed a more clinically noticeable color change, thus a more effective bleaching. Reduced values in color coordinate b* indicates a tendency of tooth to become less yellow (b* positive), showing the bleaching efficacy that was also greater for CP 20% and HP 38%. Bleaching effectiveness can also be attested by increase in tooth lightness (13), as observed in this study, all products increased the brightness of dental specimens and these did not present a significant difference in ΔL.

Although a previous study (25) did not find an increase in HP bleaching effectiveness above 15%, in this study, the HP bleaching agents 9.5% and CP 45% (~15%) showed moderate bleaching, but slightly lower compared to HP 38% and CP 20% (~6.3%). The latter even with a HP concentration lower than the first two caused a greater color change, probably due to longer time of contact with the teeth during the bleaching treatment. Therefore, the null hypothesis was not accepted.

## Conclusions

Bleaching agents showed bleaching effectiveness, promoting visual color change, increased lightness, and a tendency to decrease the yellowish tone of tooth, but with reduced hardness, which could be reversed by saliva after bleaching treatment.
